# Selecting Genetic Variants and Interactions Associated with Amyotrophic Lateral Sclerosis: A Group LASSO Approach

**DOI:** 10.3390/jpm12081330

**Published:** 2022-08-19

**Authors:** Sofia Galvão Feronato, Maria Luiza Matos Silva, Rafael Izbicki, Ticiana D. J. Farias, Patrícia Shigunov, Bruno Dallagiovanna, Fabio Passetti, Hellen Geremias dos Santos

**Affiliations:** 1 Instituto Carlos Chagas, Fundação Oswaldo Cruz, Curitiba 81310-020, Brazil; 2 Department of Statistics, Universidade Federal de São Carlos, São Carlos 13565-905, Brazil; 3 Division of Biomedical Informatics, Department of Immunology and Microbiology, University of Colorado School of Medicine, Aurora, CO 80045, USA

**Keywords:** amyotrophic lateral sclerosis, genome-wide association studies, group LASSO regularization, single-nucleotide polymorphisms, pairwise interaction

## Abstract

Amyotrophic lateral sclerosis (ALS) is a multi-system neurodegenerative disease that affects both upper and lower motor neurons, resulting from a combination of genetic, environmental, and lifestyle factors. Usually, the association between single-nucleotide polymorphisms (SNPs) and this disease is tested individually, which leads to the testing of multiple hypotheses. In addition, this classical approach does not support the detection of interaction-dependent SNPs. We applied a two-step procedure to select SNPs and pairwise interactions associated with ALS. SNP data from 276 ALS patients and 268 controls were analyzed by a two-step group LASSO in 2000 iterations. In the first step, we fitted a group LASSO model to a bootstrap sample and a random subset of predictors (25%) from the original data set aiming to screen for important SNPs and, in the second step, we fitted a hierarchical group LASSO model to evaluate pairwise interactions. An in silico analysis was performed on a set of variables, which were prioritized according to their bootstrap selection frequency. We identified seven SNPs (*rs16984239*, *rs10459680*, *rs1436918*, *rs1037666*, *rs4552942*, *rs10773543*, and *rs2241493*) and two pairwise interactions (*rs16984239:rs2118657* and *rs16984239:rs3172469*) potentially involved in nervous system conservation and function. These results may contribute to the understanding of ALS pathogenesis, its diagnosis, and therapeutic strategy improvement.

## 1. Introduction

Amyotrophic lateral sclerosis (ALS) is a multisystem neurodegenerative disease that affects both upper and lower motor neurons, causing progressive loss of muscle strength and paralysis [1,2]. Its phenotype is heterogeneous, as is the disease progression and median survival time after onset, possibly as a combination of genetic, environmental, and lifestyle risk factors [3,4]. Most ALS cases occur in adult life, near 55 years of age, predominantly among men, and have no relation to familial history. Currently, there are no reliable molecular biomarkers that enable the screening or early diagnosis of ALS, which is dependent on the clinical manifestation of the disease, which takes place when a great number of motor neurons have already been affected [5]. The El Escorial revised criteria [6] are widely used for patient classification according to different levels of diagnostic certainty, namely suspected, possible, probable, and definite ALS, mainly to ensure uniformity in clinical trials. Complementary investigation can be conducted through electrophysiological study and neuroimaging [6]. Regarding pharmacological treatment, available options contribute to increased short-term survival and reduced paralysis rate but are not effective in stopping or reversing the progression of the disease [5].

Studies dedicated to understanding the genetic characteristics of the disease can contribute not only to the comprehension of its etiology but also to the development of diagnostic tests and therapies. Genome-wide association studies (GWAS) are the most common approach to detecting relationships between genetic variants (frequently, a single-nucleotide polymorphism—SNP) and disease occurrence [7]. Since SNP data sets imply high-dimensional scenarios, where the number of variables is much larger than the sample size, a genetic model to identify genotype-phenotype relationships will be over-parameterized. For this reason, the association of each SNP with the disease is tested individually in most cases [8].

Analyses focused on one genetic variant at a time lead to a great number of simultaneous hypothesis tests, requiring control for family-wise error rate or false-discovery rates, such as Bonferroni and Benjamin–Hochberg, respectively, although the former is too conservative and the latter, less stringent [9], both heavily reduce findings with statistical significance. Additionally, the assessment of individual SNP effects does not support the detection of SNPs that are dependent on genetic interactions [7]. The body of evidence on the role of main and interaction effects in ALS genetic architecture is still under development because analyzing multiple variables at once or including genetic interactions in genotype-phenotype association models entails challenges related to the computational complexity and scalability of the methods, as well as with the accuracy and interpretability of the results.

We aim to overcome these issues by iteratively applying a two-step group LASSO procedure to select SNPs and pairwise interactions in a case-control ALS study. This approach has additional benefits for high-dimensional data composed of multi-level factors and a binary response since group LASSO makes it possible to encode each level of a factor or a pairwise interaction using a dummy variable and generating coefficients with direct interpretation [10]. By iteratively incorporating data perturbation into the analysis, using bootstrap samples together with a random subset of the predictors from the original data set, it is possible to deal with highly correlated variables [11] and derive a measure of importance for both individual SNPs and pairwise interactions [10]. Finally, by analyzing many variables simultaneously through regularized models, it is possible to consider potential association structures between variables, such as pairwise interactions between SNPs located in different genome regions.

The manuscript is organized as follows: first, we review the logistic regression models, LASSO and group LASSO methodologies, as well as pairwise interaction models with and without regularization. Then, we describe the implementation of our iterative approach, presenting the main results regarding variables and pairwise interaction selections, as well as their biological implications.

## 2. Materials and Methods

### 2.1. Data Set

We analyzed SNP data from ALS patients and controls from the National Institute of Neurological Disorders and Stroke Repository. The data set is available for download from the database of Genotypes and Phenotypes (dbGaP). Its first version includes genotype measurements of 555,352 SNPs from 276 individuals from the United States of America diagnosed with sporadic ALS (Supplementary Table S1) and 268 neurologically healthy controls [12]. Only patients classified as having possible, probable, and clinically probable laboratory-supported or definite ALS, according to El Escorial criteria [6], and those without a reported family history of ALS, were included in the study, as described by Schymick et al. (2007) [12]. Detailed information on data acquisition is available in the declaration section.

In our analysis, the response variable was represented by the presence or absence of ALS, i.e., Y∈{0,1} and each SNP refers to a polymorphic genetic locus characterized by two possible alleles, conventionally referred to as ${}^{\prime }A^{\prime }$ and ${}^{\prime }a^{\prime }$. An individual’s genotype at a marker, in turn, is denoted by the pair of alleles at a location and is represented as a three-level factor with possible values {AA,Aa,aa} [13]. We recode SNPs according to the number of minor alleles, i.e {0,1,2}, representing those alleles least frequent in the data set analyzed.

### 2.2. Genetic Model

A logistic regression model can be used to develop a genetic model aiming to understand how a predictor, i.e, an SNP, is associated with a qualitative phenotype on a genome-wide scale. Given a collection of *N* observations, a response variable, *Y*, and a set of predictors, $X=(X_{1},X_{2},$…$,X_{p})$, the general form of this model can be represented by

$$\begin{array}{c} \log\frac{P(Y=1|X)}{P(Y=0|X)}=\beta_{0}+X^{T}\beta , \end{array}$$

where $\beta_{0}\in R$ is an intercept term, and $\beta \in R^{p}$ is the vector of regression coefficients. A transformed version of P(Y=1|X) is given by the following model:

$$\begin{array}{c} P\left(Y=1|X\right)=\frac{e^{\beta_{0}+X^{T}\beta }}{1+e^{\beta_{0}+X^{T}\beta }}. \end{array}$$

The unknown coefficients $\beta_{0}$ and $\beta =(\beta_{1},$…$,\beta_{p})$ are then estimated by maximizing the likelihood or, equivalently, by minimizing the negative log-likelihood function. For *N* observations, the log-likelihood function is given by:

$$\begin{array}{c} l\left(\beta_{0},\beta \right)=\sum_{i=1}^{N}\left[y_{i}\left(\beta_{0}+x_{i}^{T}\beta \right)-\log\left(1+e^{\beta_{0}+x_{i}^{T}\beta }\right)\right] \end{array}$$

In a high-dimensional scenario, where the number of predictors, *p*, is much larger than the sample size, *N*, a relatively small number of predictors is expected to be associated with the response variable. Regularization methods can be used to modify the log-likelihood function in order to shrink to 0 those coefficients related to predictors that do not play an important role in the disease occurrence, resulting in a smaller subset of predictors [14].

### 2.3. LASSO

The least absolute shrinkage and selection operator (LASSO) is a popular technique for variable selection in high-dimensional scenarios [15]. This approach is applied to fitting models based on minimizing a $L_{1}$ regularized version of the negative log-likelihood function [13],

$$\begin{array}{c} \underset{\left(\beta_{0},\beta \right)}{minimize}\left\{-\frac{1}{N}l\left(\beta_{0},\beta \right)+{\lambda ||\beta ||}_{1}\right\}, \end{array}$$

where λ is a positive constant that controls the amount of regularization, and consequently, the number of selected predictors [14].

### 2.4. Group LASSO

In our application, we have a large number of multi-level factors as predictors, whose contribution to the linear model can be expressed through *G* groups of dummy variables, $Z_{g}\in R^{L_{g}}$, g=1,…,G, where $L_{g}$ indicates the number of levels for a particular factor *g*. From this representation, a model for P(Y=1|Z) involving *G* group variables $Z=(Z_{1},$…$,Z_{G})$ will take the form

(1) $$\begin{array}{c} P\left(Y=1|Z\right)=\frac{e^{\theta_{0}+\sum_{g=1}^{G}Z_{g}^{T}\theta_{g}}}{1+e^{\theta_{0}+\sum_{g=1}^{G}Z_{g}^{T}\theta_{g}}}, \end{array}$$

where $\theta_{0}$ is an intercept term and $\theta_{g}\in R^{L_{g}}$ is the vector of coefficients for the *g*th group. In such settings, for *N* observations, the maximum likelihood estimators of the coefficients $\theta_{0}$ and $\theta =(\theta_{1},$…$,\theta_{G})$ are obtained by minimizing the negative of the following log-likelihood function

$$\begin{array}{c} l\left(\theta_{0},\theta \right)=\sum_{i=1}^{N}\left[y_{i}\left(\theta_{0}+\sum_{g=1}^{G}z_{ig}^{T}\theta_{g}\right)-\log\left(1+e^{\theta_{0}+\sum_{g=1}^{G}z_{ig}^{T}\theta_{g}}\right)\right], \end{array}$$

subject to the restriction $\sum_{j=1}^{L_{g}}\theta_{g}^{j}=0\;\;\forall \;\;g$.

Aiming at selecting variables represented by groups of dummy variables, it is desirable to jointly select or omit all the coefficients within a group. Therefore, instead of using LASSO, which may select individual dummies rather than the entire factor [14], a group LASSO approach [16,17] can be applied to estimate ${\hat{\theta }}_{g}$ as the solution to the minimization of the negative log-likelihood function over sums of $L_{2}$-penalties:

$$\begin{array}{c} \underset{\left(\theta_{0},\theta \right)}{minimize}\left\{-\frac{1}{N}l\left(\theta_{0},\theta \right)+\lambda \sum_{g=1}^{G}\left|\right|\theta_{g}{\left|\right|}_{2}\right\}, \end{array}$$

where $\left|\right|\theta_{g}{\left|\right|}_{2}$ is the Euclidean norm of the vector $\theta_{g}$, assuming all groups will be equally penalized [17].

The $L_{2}$-norm ensures a sum-to-zero constraint in the dummy variable coefficients representing a multi-level factor, and the group variable selection is dictated by the penalty parameter (λ) that regularizes the sum over group coefficients. Since our data set is composed only of multi-level factors, we applied the group LASSO algorithm to select a candidate set of SNPs associated with the ALS phenotype.

### 2.5. Pairwise Interaction Model

To evaluate not only main effects but also interactions between any two group variables, $Z_{g}$ and $Z_{h}$, we modified the model presented in (1) by including interaction terms, $Z_{g:h}=Z_{g}\times Z_{h}$. In doing so, we have

$$\begin{array}{c} P\left(Y=1|Z\right)=\frac{e^{\theta_{0}+\sum_{g=1}^{G}Z_{g}^{T}\theta_{g}+\sum_{g<h}Z_{g:h}\theta_{g:h}}}{1+e^{\theta_{0}+\sum_{g=1}^{G}Z_{g}^{T}\theta_{g}+\sum_{g<h}Z_{g:h}\theta_{g:h}}}. \end{array}$$

and the log-likelihood function

$$\begin{array}{ccc} l(\theta_{0},\theta ) & = & \sum_{i=1}^{N}\{y_{i}\left(\theta_{0}+\sum_{g=1}^{G}z_{ig}^{T}\theta_{g}+\sum_{g<h}Z_{g:h}\theta_{g:h}\right)\\ & - & \log\left[1+\exp\left(\theta_{0}+\sum_{g=1}^{G}z_{ig}^{T}\theta_{g}+\sum_{g<h}Z_{g:h}\theta_{g:h}\right)\right]\} \end{array}$$

subject to the following restrictions for main and pairwise interaction effects, respectively: $\sum_{j=1}^{L_{g}}\theta_{g}^{j}=0\;\;\forall \;\;g$, and $\sum_{j=1}^{L_{g}}\theta_{g:h}^{jk}=\sum_{k=1}^{L_{h}}\theta_{g:h}^{jk}=0\;\;\forall \;\;j,k$ and g<h.

### 2.6. Hierarchical Group LASSO Regularization

Variable selection using the group LASSO method described previously can be extended to incorporate pairwise interactions. Thus, the model is fitted by minimizing:

$$\begin{array}{c} \underset{\left(\theta_{0},\theta \right)}{minimize}\left\{-\frac{1}{N}l\left(\theta_{0},\theta \right)+\lambda \left(\sum_{g=1}^{G}\left|\right|\theta_{g}{\left|\right|}_{2}+\sum_{g<h}Z_{g:h}\left|\right|\theta_{g:h}{\left|\right|}_{2}\right)\right\} \end{array}$$

In our application, we considered all pairwise interactions within the selected candidate set of SNPs through a logistic regression pairwise interaction model via hierarchical group LASSO regularization [17].

### 2.7. Implementation

Based on variable selection procedures described in the random LASSO [11], recursive random LASSO [18], and high-dimensional LASSO [19] approaches, we performed 2000 iterations of a two-step analysis to search for SNPs and pairwise interactions associated with the ALS phenotype (Supplementary Figure S1).

As the first step of each iteration, we used a bootstrap sample and a random subset of the predictors (25%) (candidate variables) from the original data set to fit a group LASSO model aiming to screen for important SNPs, thus restricting the pairwise interaction search space. In the second step, we applied the hierarchical group LASSO regularization method in order to consider pairwise interactions between all the selected predictors.

Both group LASSO and hierarchical group LASSO regularization were computed at 50 λ values. The best value for this parameter was obtained using a 10-fold cross validation process. We chose the largest value of λ whose cross-validation error was within one standard error of the minimum, which was then used to fit the model for the corresponding iteration [13].

Next, we describe how the threshold for variable selection in the first step was defined. Since we randomly selected 25% of predictors to adjust a group LASSO in each iteration, the probability of a SNP, $Z_{g}$, g=1,2,…,G, to be included (I) in the analysis is P(I)=0.25 and the expected number of inclusions of $Z_{g}$ in 2000 iterations is 0.25×2000=500 (Supplementary Figure S2). The number of selections for each predictor, in turn, will depend not only on its degree of association with the response variable but also on the other predictors considered in the model fit. Therefore, those SNPs with a selection frequency of 70% or more, i.e., approximately 350 selections in 500 inclusions, were prioritized for the functional analysis.

Regarding pairwise interactions, according to our approach, an interaction will be evaluated only for SNPs that were jointly selected in the first step. The probability of any two SNPs, $Z_{g}\cap Z_{h}$, g,h=1,…,G and g<h, to be included in the first step is $P\left(I_{Z_{g}\cap Z_{h}}\right)=P\left(I\right)\;\cdot \;P\left(I\right)=0.25\;\cdot \;0.25=0.0625$. Thus, the expected number of $Z_{g}\cap Z_{h}$ inclusions in 2000 iterations is 0.0625·2000=125 (Supplementary Figure S3). The number of selections of any two SNPs will depend on their effects given the other predictors in the model. Thus, we were more permissive in choosing cut-off points in this context. For any SNPs jointly selected at least 50% of the time in the first step, i.e., approximately 60 selections in 125 inclusions, we evaluated the frequency of pairwise interaction selection in the second step. Pairwise interactions with a selection frequency of at least 50%, that is, 30 selections in 60 inclusions in the interaction search model, were considered for the functional analysis.

### 2.8. Descriptive and Analytical Statistics

For prioritized SNPs and pairwise interactions, we estimated the crude odds ratio (OR) and the correspondent 95% confidence interval (95%CI) by adjusting a logistic regression model on the original data. The log-likelihood ratio test (LRT) was used to evaluate the significance of the selected interactions. A *p*-value <0.05 was considered statistically significant for the logistic regression model and the LRT. The statistical analysis was repeated for the following ALS disease subgroups, defined according to (1) El Escorial criteria [6]—definite or probable/laboratory probable; (2) site of symptom onset—bulbar or limb; (3) age at symptom onset—less than or equal to 45 years or more than or equal to 65 years. All ALS disease subgroups were compared with the control group (n = 271).

Descriptive results from the iterative process, such as the number of selected SNPs in each iteration and group LASSO estimated coefficients for the prioritized SNPs are presented. Analyses were performed using the R software, and the model fit was determined through group LASSO penalized learning using a unified blockwise-majorization-descent algorithm (gglasso) and learning interactions via hierarchical group LASSO regularization (glinternet) packages.

### 2.9. In Silico Analysis

We annotated the prioritized SNPs, both on the first and second steps, with UCSC [20] and Ensembl [21] genome browsers. The possible structural and regulatory impacts of these genetic variants and their expression quantitative trait loci (eQTL) effects were identified from HaploReg [22], RegulomeDB [23], LDlink [24], dbSNP [25], GTEx Portal [26], and Protein atlas [27] web tools.

## 3. Results and Discussion

We performed quality control (QC) on genotype data to include SNPs with complete genotyping call rate, minor allele frequency >5% and Hardy–Weinberg equilibrium *p*-value < $1\;\cdot \;10^{-6}$ for controls. In addition, we pruned SNP panels based on linkage disequilibrium via sliding windows, according to PLINK software implementation (a window size in variant count equal to 10, step size equal to 10, corresponding to a variant count to shift the window, and threshold based on correlations between genotype allele counts equal to 0.80). After genotype QC, the data set comprised 254,293 SNPs (group variables) for variable selection. From those, 252,252 SNPs were represented by three dummy variables (i.e., those presenting the three genotypes), and 2041 SNPs by two dummies.

### 3.1. Variable Selection

The distribution of the number of selected variables in 2000 iterations is shown in Supplementary Figure S4. The corresponding minimum, median, and maximum values were 141, 187, and 226 SNPs, respectively. From 254,293 variables, 79% were never selected when included in the group LASSO model, reinforcing its sparseness, and the hypothesis that only a small set of SNPs is important in explaining the outcome of interest [13].

**Figure 1:**
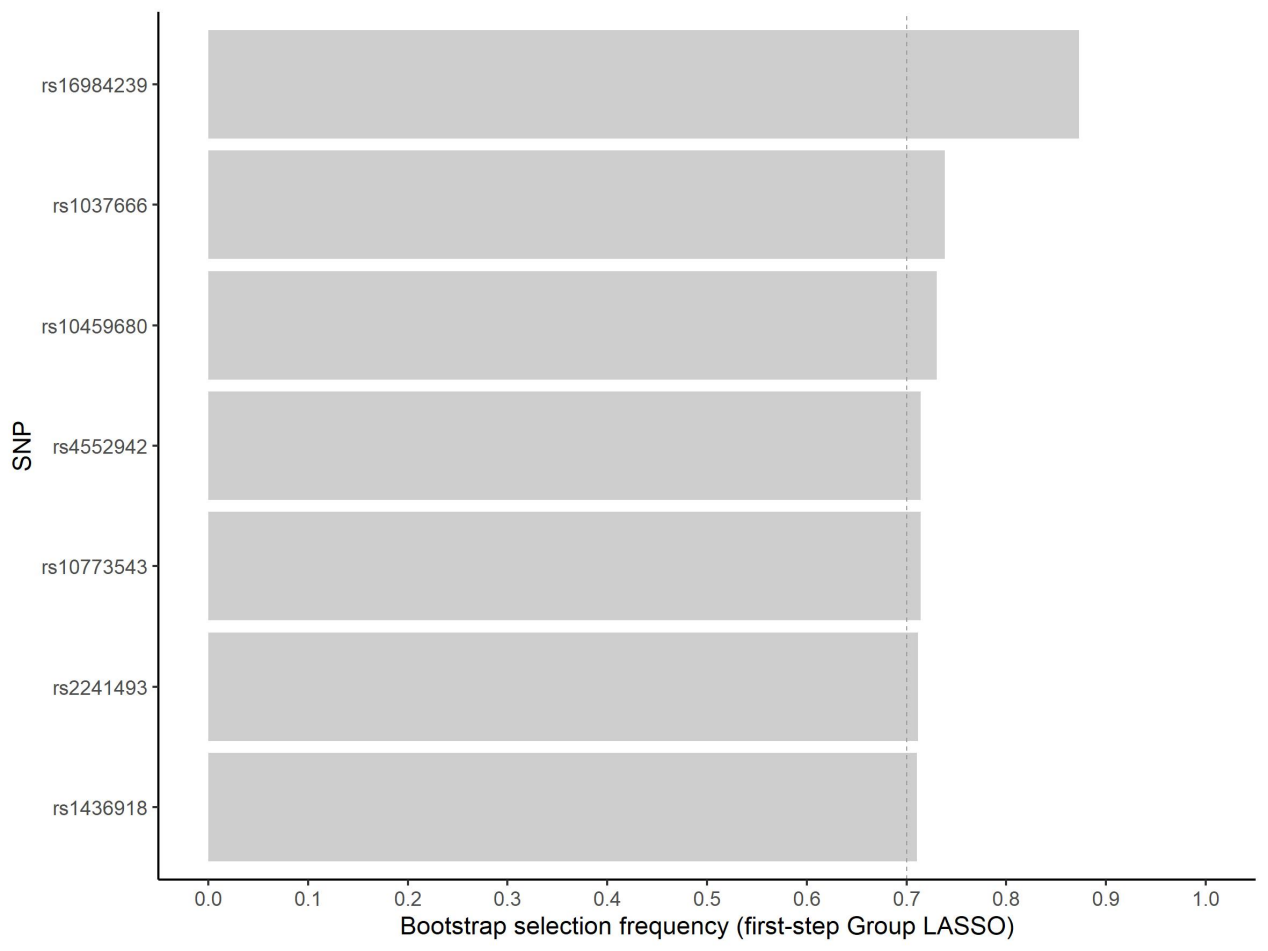
Frequency of non-zero estimated coefficient for the seven most frequently selected variants in the first step group LASSO regularization.

Variable selection reduces the computational burden for pairwise interaction searches. Bootstrap analysis, in turn, can be applied to assess the stability of selected variables, contributing to variable importance evaluation [28]. We prioritized seven variables for the in silico analysis (Figure 1). For these variables, the minimum, median and maximum number of selections in approximately 500 inclusions in model fit were 345, 358, and 412, respectively.

**Figure 2:**
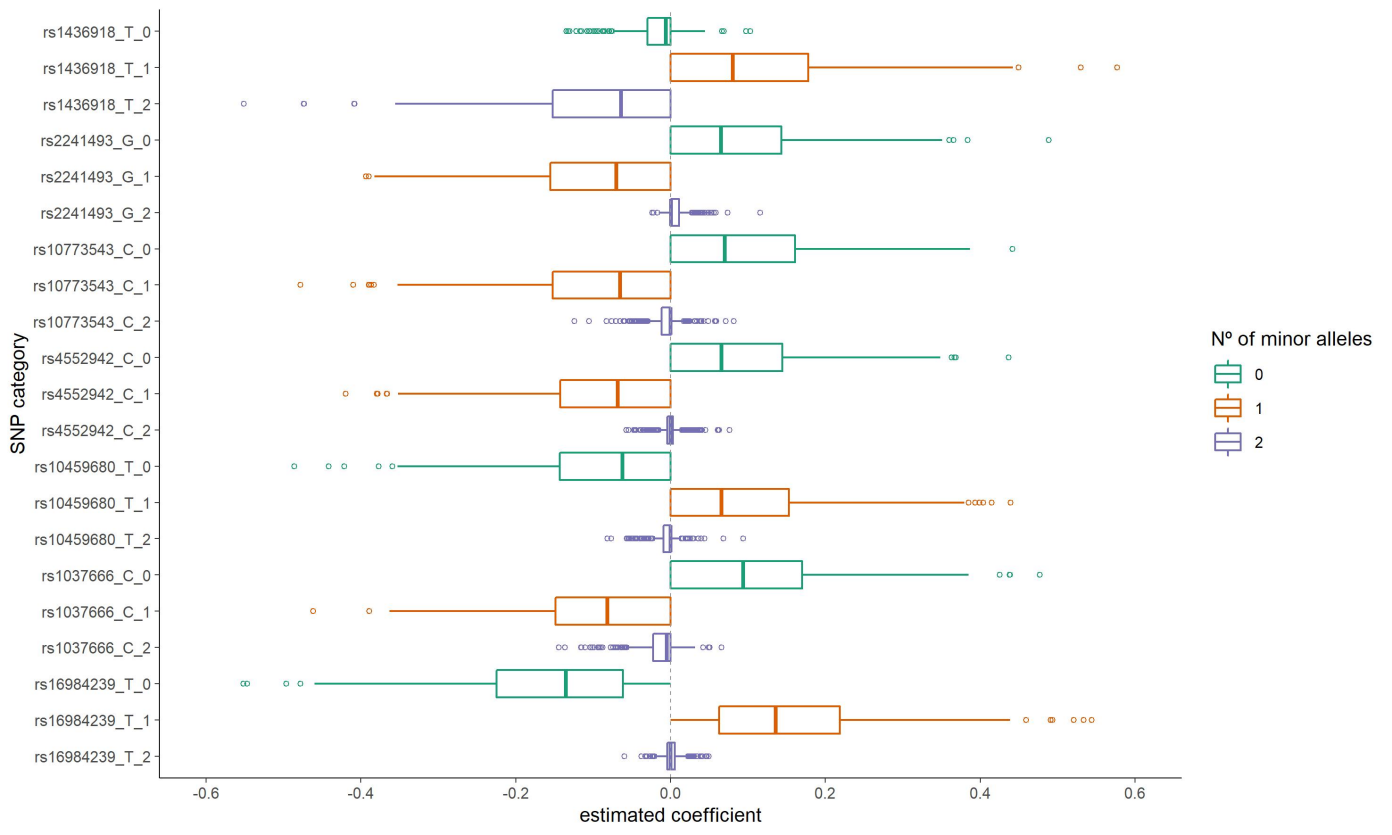
Distribution of the estimated coefficients for the seven most frequently selected variants in the first step group LASSO regularization.

The distribution of the group LASSO estimated coefficients for the categories of these SNPs is presented in Figure 2. For three of them (*rs16984239*, *rs10459680*, and *rs1436918*), having one copy for the minor allele resulted in a positive coefficient estimate (increased odds of ALS). On the other hand, for the SNPs *rs1037666*, *rs4552942*, *rs10773543*, and *rs2241493* having one copy for the minor allele presented a negative coefficient. Additionally, for *rs1037666* and *rs1436918* SNPs, having two copies for the minor allele also resulted in a negative coefficient. It is important to note that these estimates are adjusted for other SNPs considered together in the model fit in the corresponding iteration.

Table 1 shows the estimated crude odds ratio (OR) for these seven SNPs according to the original data set, revealing results similar to those presented in Figure 2. Briefly, considering the absence of minor allele copies as our reference category, the presence of one copy for the minor alleles *rs16984239 A* (OR = 2.74; 95%CI: 1.85;4.10), *rs10459680 T* (OR = 2.23; 95%CI: 1.56;3.19), and *rs1436918 A* (OR = 1.63; 95%CI: 1.07;2.48) can increase susceptibility to ALS. On the other hand, carrying *rs4552942 C* (OR = 0.45; 95%CI: 0.31;0.64), *rs10773543 G* (OR = 0.44; 95%CI: 0.31;0.64), *rs2241493 G* (OR = 0.44; 95%CI: 0.30;0.64) and *rs1037666 C* (OR = 0.45; 95%CI: 0.31;0.64) single alleles, as well as *rs1037666 CC* (OR = 0.53; 95%CI: 0.29;0.95) and *rs1436918 AA* (OR = 0.59; 95%CI: 0.36;0.98) genotypes, can decrease susceptibility to ALS.

The effect of many genetic variants on the occurrence of the disease in humans is currently unknown. However, any variant can interfere with normal biological function and cause disease at different levels of severity [29]. Thus, we characterized the selected SNPs according to (1) their position in the genome; (2) their genomic context—genes and nature of the region (Table 2), and (3) their potential biological implications. We also searched for previous studies relating the SNP to ALS susceptibility. Below, we detail the main functional characteristics of the regions in which these SNPs are located.

In a review of the molecular and cellular mechanisms involved in the pathogenesis of ALS, Le Gall et al. (2020) [43] highlighted the following pathways: oxidative stress, mitochondrial dysfunction, axonal transport, glutamate excitotoxicity, endosomal and vesicular secretions, protein homeostasis, and RNA metabolism. The authors emphasized the relationship between defects in these pathways, which are responsible for exacerbating disruption of cellular homeostasis and, consequently, microglial activation, neuroinflammation, astrocytosis, motor neuron death, and muscle denervation.

Our analysis revealed two SNPs related to genes in the biological process of ion transport, *rs16984239* and the *rs2241493*. The former occurs in a genomic region near the *potassium voltage-gated channel modifier subfamily S member 3 (KCNS3)* gene. This SNP was also highlighted by other authors who analyzed the ALS SNP data set provided by Schymick et al. (2007) [12], as described in Table 2. The latter is a missense polymorphism affecting a protein encoded by the *transient receptor potential cation channel subfamily M member 1 (TRPM1)* gene. Additionally, this SNP induces a substitution of serine (AGC) amino acid by isoleucine (ATC), threonine (ACC), or asparagine (AAC) according to the dbSNP database [25] (Supplementary Figure S5), which might imply variations in pKa levels, leading to different electrical states depending on the pH of the medium.

The *KCNS3* gene has a widespread tissue distribution [44] but is highly expressed in the lungs, according to the GTex Portal [26]. The main functions of voltage-gated potassium channels are the resting membrane potential regulation and the shape and frequency control of action potentials. The KCNS3 protein is not functional by itself but modulates the activation and deactivation rates of other potassium voltage-gated channel proteins [45]. The *TRPM1* gene is highly expressed in testicular and skin tissues, according to the GTex Portal [26] and encodes a protein that forms non-selective divalent cation-conducting channels responsible for membrane depolarization [27]. This gene plays a role in visual pathways and is expressed in retina center-ON bipolar neurons and melanocytes [46].

In addition, two SNPs associated with actin cytoskeleton organization were prioritized for the in silico analysis. The *rs1037666* is located in the intron 4 of the *Formin-2 (FMN2)* gene, which has an actin-binding molecular function, and the *rs10773543* is located within intron 2 of the transmembrane protein 132C *(TMEM132C)* gene.

The FMN2 protein plays an important role in the organization of the actin cytoskeleton and in cell polarity [27]. It is highly expressed in the fetal brain and in all tissues of the adult central nervous system, acting on cytoskeletal processes during axonal growth, migration, and synapse formation [47]. As suggested by Law et al. (2014) [48], this protein seems to regulate actin cytoskeleton formation during spinal development, maturation, or remodeling, with implications for neuronal functions that mediate higher cognition in humans. In addition, Mutalik (2018) [49] explored the role of FMN2 in the organization of actin structures in neuronal growth cones, demonstrating its essential function in neuronal regeneration.

Transmembrane proteins are responsible for maintaining cell junctions in the central nervous system. The TMEM132 family of proteins has a cell adhesion function, connecting the extracellular environment with the intracellular actin cytoskeleton, thus playing an important role in the regulation of changes in the morphology, motility, and migration of neuronal cells [50]. However, the specific role of TMEM132C is still poorly understood.

Two other selected SNPs are located in intronic regions. The *rs10459680* SNP is located within intron 4 of the *LOC101927025* non-coding gene, and is genomically close to the *repulsive-guidance molecule (RGM) bone morphogenetic protein (BMP) co-receptor A (RGMA)* gene, according to the HaploReg webtool [22]. The RGMA protein is a member of the RGM family that plays several roles in the central nervous system, such as neural tube closure, neurite outgrowth, cortical neuron branching inhibition, and mature synapse formation [27]. In particular, RGMA regulates repulsive axonal guidance and neuronal survival via neogenin-1 binding [51], and alterations in its expression and function have been previously related to central nervous system diseases, such as multiple sclerosis, spinal cord injury, and Parkinson’s disease [52].

The *rs4552942* SNP is located within intron 1 of the *long intergenic non-protein coding RNA 2055 (LINC02055)* gene, according to the Ensembl genome browser [21]. This long non-coding RNA has a diversified distribution, with high expression in testis and frontal cortex tissues, as indicated by the GTEx portal [26]. We could not find any literature regarding the biological implications of the *LINC02055* gene. Nonetheless, by analyzing the ALS SNP data set provided by Schymick et al. (2007) [12], Sha et al. (2009) [30] prioritized the *rs12680546* SNP as an interaction pair (*p*-value = 0.156), which in turn is in high linkage disequilibrium with *rs4552942* ($r^{2}=0.976$).

We also prioritized the *rs1436918* SNP, which is within a regulatory region genomically close to the *golgin A8 family member B (GOLGA8B)* gene, according to the HaploReg webtool [22]. The *GOLGA8B* gene is a member of the Golgin family and is part of the Golgin matrix [53]. It is highly expressed in thyroid and brain tissues, mainly in the cerebellum and the cerebellar hemisphere tissue. Interestingly, the *rs1436918 GG* genotype was associated with increased *GOLGA8B* expression in skeletal muscle, according to eQTL expression level analysis [26]. Despite the known histological evidence of Golgi apparatus fragmentation in motor neurons of ALS patients [54,55], the GOLGA8B protein has not yet been described as related to ALS pathogenesis.

### 3.2. Pairwise Interaction Selection

A genetic interaction refers to any type of interaction between segments of the genome, which can occur at different levels of the biological system, from the direct relation of genes to the physical interaction of proteins and the physiological interaction of different metabolic pathways, all of which can have beneficial or harmful implications for the overall expression of a phenotype [56].

**Figure 3:**
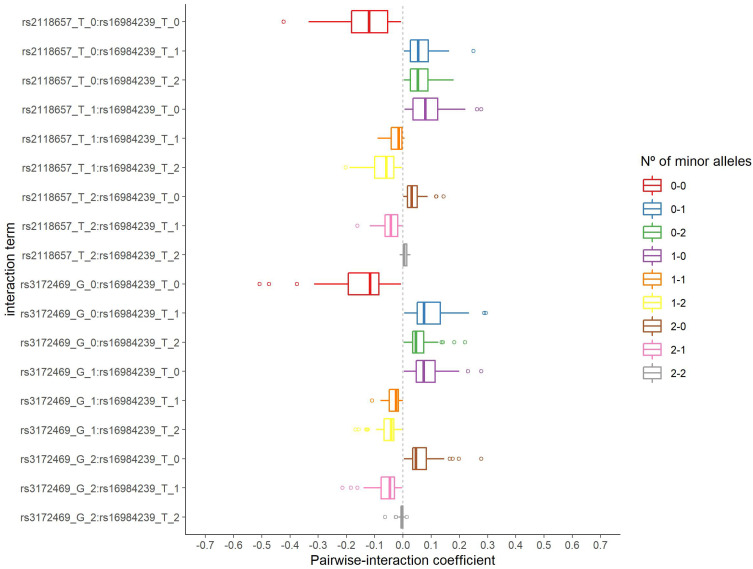
Distribution of the estimated coefficients for the two pairwise interactions selected in the second step group LASSO regularization.

The distribution of the number of selected pairwise interactions in 2000 iterations is presented in Supplementary Figure S6. The corresponding minimum, median, and maximum values were 141, 187, and 226 pairwise interactions, respectively. The *rs16984239:rs2118657* and *rs16984239:rs3172469* interactions were both selected 37 times from 58 and 69 s step model fits, respectively, and were considered for the in silico analysis. Figure 3 shows the distribution of the estimated coefficient for each category of these pairwise interactions. They have a similar pattern, with negative coefficients for the absence of the minor allele on both SNPs and coefficients of approximately zero for two minor alleles on both SNPs.

Table 3 presents the relationship of the *rs16984239* with ALS in the strata of its interaction pairs, based on the original data set. Such analysis was performed to describe the effect of each genotype combination on the odds of having ALS. Considering the absence of the minor allele as the reference category, the *rs16984239 A* allele can increase ALS susceptibility in the following strata: both *rs2118657* (OR = 4.12; 95%CI:2.38;7.13) and *rs3172469* (OR = 4.08; 95%CI:2.37;7.02) absence of the minor allele, and *rs2118657 T* (OR = 1.90; 95%CI:1.01;3.55) and *rs3172469 G* (OR = 1.96; 95%CI:1.04;3.71) single alleles.

In addition, the *rs16984239 AA* genotype showed greater odds of ALS in the absence of the minor allele for *rs2118657*, albeit with a wide 95% confidence interval, due to the small sample size for this combination of genotypes (OR = 12.59; 95%CI:1.48;106.89). The result was in the opposite direction for the *rs2118657 T* single allele stratum, although it was not statistically significant (OR = 0.15; 95%CI:0.02;1.30). Finally, as the presence of two minor allele genotypes was a rare event, there were no individuals in the category corresponding to this combination of genotypes for both interactions analyzed.

We applied a likelihood ratio test (LRT) to evaluate these interactions. Only the pairwise interaction *rs16984239:rs2118657* was statistically significant (*p*-value = 0.00214). For the pairwise interaction *rs16984239:rs3172469*, the observed LRT *p*-value was 0.21060. It should be emphasized that, although not statistically significant, when the interaction results in markedly different estimates (in both magnitude and direction) for the association between a factor and a response variable in the strata specified by its interaction pair, the possibility of a true interaction effect is reinforced. However, such interactions should be evaluated by a study with sufficient statistical power, including a discussion in regard to their biological plausibility [57].

Both the *rs2118657* and *rs3172469* SNPs are located in chromosome 3 (linkage disequilibrium $r^{2}<0.05$). *rs2118657* is intergenic, genomically close to the *Butyrylcholinesterase (BCHE)* gene, which originated from an ancient duplication of acetylcholinesterase *(ACHE)* in vertebrate evolution [58]. The cholinesterase enzymes are expressed by astrocytes and play an important role in choline-based neurotransmitter degradation. Thus, by preventing synaptic overstimulation, BCHE may be important for neuroprotection, both in the brain and in the neuromuscular junction [58]. Reduced BCHE function can, therefore, lead to the accumulation of neurotransmitters in the synapses, such as glutamate, implying astrocyte toxicity [59]. The glutamate excitotoxicity pathway is well-established in ALS pathogenesis as a consequence of defects in glutamate transport and uptake [43]. Additionally, previous research explored the association between astrocyte toxicity, motor neuron degeneration, and loss of muscle coordination in ALS patients [60].

The *rs16984239:rs2118657* interaction pair could represent a relationship between BCHE-KCNS3 proteins, implying a cholinesterase–potassium balance involved in synapse function. BCHE is an important cholinergic regulator present in neurons and motor endplates, which are also rich in potassium-gate regulators, such as KCNS3. Glial protection against neurotoxicity was previously related to potassium balance control [61]. Thus, intracellular potassium imbalance, caused by improper KCNS3 regulation, might lead to cholinesterase inhibition, astrocyte toxicity, and, ultimately, motor neuron degeneration.

The *rs3172469* SNP is located in intron 1 of the *B-cell lymphoma 6 (BCL6)* gene, and encodes a transcriptional repressor that may be involved in the modulation of several metabolic pathways, the most popular being related to B cell activation implied in lymphoma pathogenesis [62]. The *BCL6* gene is highly expressed in whole blood, musculoskeletal, and tibial nerve tissues, according to the GTEx Portal [26], and was previously described as a specific gene to spinal motor neurons [63].

The *rs16984239:rs3172469* pair could, therefore, be related to a BCL6-*KCNS3* protein-gene association, particularly due to the transcriptional repression capabilities of the BCL6 protein and its known presence in spinal motor neurons [63]. Wang et al. (2006) [64] reported a down-regulated expression of BCL6 in ALS post-mortem tissues. Regulatory processes of the BCL6 protein in the *KCNS3* gene might imply over-expression of KCNS3, leading to an overstimulation of neuronal synapses, which may contribute to the general loss of coordination and muscle tremor symptoms in the ALS phenotype [1,65].

To assess whether the variability in clinical findings can modify the odds of ALS observed for the prioritized SNPs and pairwise interactions, we evaluated the association between these variables and the presence of ALS in subgroups defined according to El Escorial criteria, as well as age and site of symptom’s onset. In general, the estimated odds ratios showed the same direction as those observed considering the ALS cases together, but with differences in magnitude and statistical significance, possibly due to the smaller sample size in each ALS disease subgroup (Supplementary Tables S2 and S3).

### 3.3. LASSO and Related Approaches in Variable Selection

Schymick et al. (2007) [12] were the first to evaluate GWAS in ALS. The authors identified 34 SNPs with an unadjusted *p*-value <0.0001 for single-marker tests. However, *p*-value correction for multiple comparisons by the Bonferroni method revealed that no SNP maintained statistical significance at the 5% level. Interestingly, six out of the seven variables we prioritized in the first step of our procedure were in this set of variables (only *rs2241493* was not highlighted). On the other hand, neither the *rs2118657* nor the *rs3172469* variants were pointed out by the authors.

Sha et al. (2009) [30] searched for pairwise interactions in the aforementioned ALS SNP data set [12]. Since an exhaustive search through all combinations of genetic variants in the complete data set would be computationally infeasible, the authors applied a two-step procedure aiming to select a set of important genetic variants associated with the ALS phenotype, thus reducing the variable space for pairwise interaction evaluation. The 1000 genetic variants with the lowest *p*-values for the single-marker test were selected and two pairwise interaction terms were identified in this set: *rs4363506:rs3733242* and *rs4363506:rs16984239*. We also selected pairwise interactions with SNP *rs16984239* on the same data set. However, this pair was not evaluated by our approach, because the *rs4363506* SNP was excluded in the quality control step (genotype rate <1). Additionally, the *rs3733242* SNP presented an estimated coefficient equal to 0 on 84% of the times it was included on the first step group LASSO fit and was rarely evaluated on the second step for pairwise interaction.

In addition to single-marker tests, two-step approaches involving multiple regression with regularization to select main [11,18,19] and pairwise interaction effects [10,66,67,68] on high-dimensional data are extremely attractive as they can accommodate correlated variables into the model fit as well as more variables than the sample size. Additionally, two-step approaches can be applied iteratively by using bootstrap samples together with random variable subsets from the original data to derive importance measures for both individual SNPs and pairwise interactions [10,11,18,19]. Finally, by incorporating the group characteristic of the genetic variants in the selection process, the group LASSO solves some disadvantages of the logistic regression model, such as the increasing number of parameters arising from three-level SNP factors and their pairwise interactions [10]. It also improves upon the conventional LASSO approach, which may select individual SNP levels rather than the entire related SNP factor [14].

Combining these different ideas, we iteratively applied the group LASSO method, both for individual SNP (first step) and pairwise interaction (second step) selection, using a cross-validation process to choose the penalty parameter for each model fit. These procedures are integrated since the variables selected in the first step according to a group LASSO fit were evaluated for all possible interactions by a hierarchical group LASSO model at each iteration. Finally, variable importance measures were calculated from the bootstrap selection frequency in 2000 iterations.

Our proposal has limitations. First, it is not possible to test all pairwise interactions in complete SNP data, and some mechanism to reduce its dimension is necessary. Thus, we randomly and iteratively included subsets of variables to fit a regularized model allowing greater variability in the selection of variables in the first step, and consequently, the evaluation of a greater number of pairwise interactions, in the second step. Nonetheless, as a random subset of variables was included in the regularization model in each iteration, a greater variability for the estimated coefficients is also to be expected, since in a regression model the effect of a variable depends on which other variables are considered together. However, the individual SNPs and the selected pairwise interactions presented consistent results for the estimated coefficients in the bootstrap analyses: even with different effect sizes, their signs were preserved, as evidenced in the distributions of the estimated coefficients shown in Figure 2 and Figure 3. Furthermore, the logistic regression fit results shown in Table 2 and Table 3 revealed patterns similar to those from bootstrap analyses.

Our criterion for establishing a final set of variables for further in silico analysis was based on an ad hoc cut-off point for their bootstrap selection frequency. Importantly, all statistical measures have drawbacks, including *p*-values from logistic regression fits, leave-one-out indices, as proposed by Wu et al. (2007) [69], and *p*-values from statistical tests applied to genetic variant selection based on bootstrap results, as proposed by Park et al. (2015) [18] and revised by Kim et al. (2019) [19]. These drawbacks are pointed out by their very proponents and mean that these measures are not ideal for determining the global statistical or biological significance of genetic variants. However, all of them, including our approach, can lead to interesting findings that may be replicated and evaluated in subsequent studies and have their biological implications investigated.

The SNP data set analyzed provides limited data regarding the clinical characteristics of ALS patients. Additionally, environmental and lifestyle factors were not investigated. It is known that ALS is a heterogeneous multi-system neurodegenerative disease [2], and its onset may result from a combination of genetic, environmental, and lifestyle factors. Recently, Hop et al. (2022) [70] reported interesting epigenetic findings regarding DNA methylated positions more expressed in ALS patients than in healthy controls, annotated to genes implicated in metabolic, inflammatory, and cholesterol pathways. Furthermore, these methylated positions overlapped with trait-associated positions related to HDL cholesterol, triglyceride concentration, body mass index, and alcohol consumption. Additionally, regarding environmental factors, an up-to-date meta-analysis confirmed the history of trauma, mainly trunk trauma, as a risk factor for ALS [71]. Future studies that incorporate these predictors may help to elucidate other aspects of the pathogenic mechanisms of ALS as well as its treatment and prevention in clinical practice.

## 4. Conclusions

We selected seven SNPs and two pairwise interactions associated with the ALS phenotype by applying a two-step group LASSO approach and described their biological consequences through in silico analysis. In summary, the biological implications of the selected genetic variants revealed proteins related to membrane potential regulation, Golgi apparatus fragmentation, actin cytoskeleton and cell polarity organization, axon guidance, and neurotransmitter metabolism. *rs2241493* is the only SNP in a coding region and is also a missense polymorphism. Therefore, *rs2241493* seems to be the most promising candidate for future functional studies. Two pairwise interactions were prioritized for in silico analyses: *rs16984239:rs2118657* and *rs16984239:rs3172469*. Although *rs16984239* has been identified by other studies that explored the same ALS SNP data we analyzed, neither *rs2118657* nor *rs3172469* was previously described as associated with the ALS phenotype. We believe our results may contribute to a better molecular understanding of the ALS phenotype, with the potential to be tested in diagnosis or in therapeutic strategy improvement.

ALS is known to be clinically and genetically highly variable, and future studies aiming to prioritize genetic variants capable of differentiating ALS subgroups can be explored using the approach proposed in the present study. Additionally, ALS is hypothesized to be related not only to genetics but also to environmental and lifestyle factors. Therefore, considering these characteristics as predictors of ALS in future studies may improve the holistic understanding of the mechanisms involved in disease pathogenesis.

## Figures and Tables

**Table 1:** Non-adjusted odds ratio for amyotrophic lateral sclerosis according to the prioritized single-nucleotide polymorphisms in the first step of the group LASSO regularization.

Variant (Minor Allele)	Total n	ALS n (%)	OR	95%CI	*p*-Value
rs16984239 (A)					
0	378	165 (0.44)	reference		
1	153	104 (0.68)	2.74	1.85; 4.10	<0.001
2	13	7 (0.54)	1.51	0.49; 4.76	0.469
rs1037666 (C)					
0	247	151 (0.61)	reference		
1	242	100 (0.41)	0.45	0.31; 0.64	<0.001
2	55	25 (0.45)	0.53	0.29; 0.95	0.035
rs10459680 (T)					
0	281	118 (0.42)	reference		
1	230	142 (0.62)	2.23	1.56; 3.19	<0.001
2	33	16 (0.48)	1.30	0.63; 2.69	0.477
rs4552942 (C)					
0	277	165 (0.60)	reference		
1	226	090 (0.40)	0.45	0.31; 0.64	<0.001
2	41	21 (0.51)	0.71	0.37; 1.38	0.313
rs10773543 (G)					
0	230	142 (0.62)	reference		
1	261	109 (0.42)	0.44	0.31; 0.64	<0.001
2	53	25 (0.47)	0.55	0.30; 1.01	0.054
rs2241493 (C)					
0	345	198 (0.57)	reference		
1	180	067 (0.37)	0.44	0.30; 0.64	<0.001
2	19	11 (0.58)	1.02	0.40; 2.70	0.966
rs1436918 (A)					
0	129	061 (0.47)	reference		
1	288	171 (0.59)	1.63	1.07; 2.48	0.022
2	127	44 (0.35)	0.59	0.36; 0.98	0.040

**Table 2:** Genome position and genomic context of the selected single-nucleotide polymorphisms.

SNP	Chr:Location	Gene	Consequence	Phenotype	Citation
**First step**					
rs16984239	2:18053180	-	intergenic	ALS	[12,30,31,32,33]
rs1037666	1:240195185	FMN2	intronic	ALS	[12]
rs10459680	15:93138241	LOC101927025	intronic	ALS	[12]
rs4552942	8:135862080	LINC02055	intronic	ALS; core binding factor acute myeloid leukemia	[12,34]
rs10773543	12:128439181	TMEM132C	intronic	ALS	[12]
rs2241493	15:31070149	TRPM1	missense	Congenital stationary night blindness, type 1C	[35,36,37,38,39]
rs1436918	15:34644720	LOC390569	regulatory genomic region	ALS	[12,40]
**Second step**					
rs2118657	3:165864723	-	intergenic	-	-
rs3172469	3:187741300	BCL6	intronic	Myeloma; non-Hodgkin lymphoma	[41,42]

**Table 3:** Non-adjusted odds ratio for amyotrophic lateral sclerosis according to the selected pairwise interactions in the second step group LASSO regularization.

Variant (Minor Allele)	Total n	ALS n (%)	OR	95%CI	*p*-Value
rs2118657 (T) = 0					
rs16984239 (A) = 0	189	61 (0.32)	reference		
rs16984239 (A) = 1	83	55 (0.66)	4.12	2.38; 7.13	<0.001
rs16984239 (A) = 2	7	6 (0.86)	12.59	1.48; 106.89	0.020
rs2118657 (T) = 1					
rs16984239 (A) = 0	162	93 (0.57)	reference		
rs16984239 (A) = 1	64	46 (0.72)	1.90	1.01; 3.55	0.046
rs16984239 (A) = 2	6	1 (0.17)	0.15	0.02; 1.30	0.085
rs2118657 (T) = 2					
rs16984239 (A) = 0	27	11 (0.41)	reference		
rs16984239 (A) = 1	6	3 (0.50)	1.45	0.25; 8.58	0.679
rs16984239 (A) = 2	-	-	-	-	-
rs3172469 (G) = 0					
rs16984239 (A) = 0	200	65 (0.33)	reference		
rs16984239 (A) = 1	83	55 (0.66)	4.08	2.37; 7.02	<0.001
rs16984239 (A) = 2	8	4 (0.50)	2.08	0.50; 8.57	0.312
rs3172469 (G) = 1					
rs16984239 (A) = 0	150	84 (0.56)	reference		
rs16984239 (A) = 1	63	45 (0.71)	1.96	1.04; 3.71	0.037
rs16984239 (A) = 2	5	3 (0.60)	1.18	0.19; 7.26	0.859
rs3172469 (G) = 2					
rs16984239 (A) = 0	28	61 (0.47)	reference		
rs16984239 (A) = 1	7	4 (0.57)	1.00	0.19; 5.33	0.999
rs16984239 (A) = 2	-	-	-	-	-

## Data Availability

We analyzed data from a case-control GWAS from the National Institute of Neurological Disorders and Stroke Repository available for download through the database of Genotypes and Phenotype (dbGaP) Authorized Access System (dbGaP study accession phs000101.v3.p1; Data Access Request number 87433-1). We made our R code available on GitHub repository https://github.com/Hellengeremias/two-step_Group_LASSO_analysis.

## Supplementary Materials

The following supporting information can be downloaded at https://www.mdpi.com/article/10.3390/jpm12081330/s1, Figure S1: Flowchart of the iterative two-step group LASSO procedure. Figure S2: Expected number of inclusions of group variables in the first step group LASSO approach, $Z_{g}$, g=1,2,…,G, in 2000 iterations. Figure S3: Expected number of inclusions in the first step group LASSO approach for any two group variables, $Z_{g}\cap Z_{h}$, g,h=1,…,G and g<h in 2000 iterations. Figure S4: Distribution of the number of selected variants in each iteration of first step group LASSO regularization. Results from 2000 iterations, each related to a group LASSO model from bootstrap samples and a 25% of available variants. Figure S5: TRPM1 protein. (A) Tertiary and secondary structure of a TRPM1 protein. Detail for the amino acid serine related to alterations caused by *rs2241493*. PDB = AF-Q7Z4N2-F1. (B) Comparison of the chemical structure of serine that is replaced by isoleucine, threonine, or asparagine in *rs2241493*. Figure S6: Distribution of the number of selected pairwise interactions in each iteration of second step group LASSO regularization. Results from 2000 iterations, each related to a group LASSO regularization model from bootstrap samples and first step selected variants. Table S1: Description of the clinical characteristics of ALS patients (n = 276). Table S2: Non-adjusted odds ratio for ALS disease subgroups (El Escorial criteria, site of symptom onset, and age at symptom onset) according to the prioritized single-nucleotide polymorphisms in the first step group LASSO regularization. All comparisons were made with the control group (n = 271). Table S3: Non-adjusted odds ratio for ALS disease subgroups (El Escorial criteria, site of symptom onset, and age at symptom onset) according to the pairwise interactions in the second step group LASSO regularization. All comparisons were made with the control group (n = 271).

## Abbreviations

The following abbreviations are used in this manuscript:

ALS	Amyotrophic Lateral Sclerosis
GWAS	Genome-Wide Association Studies
SNP	Single-Nucleotide Polymorphism
LASSO	Least Absolute Shrinkage and Selection Operator
eQTL	Expression Quantitative Trait Loci
QC	Quality Control
OR	Odds Ratio
CI	Confidence Interval
LRT	Likelihood Ratio Test
KCNS3	Potassium Voltage-Gated Channel Subfamily S member 3
FMN2	Formine-2
RGM	Repulsive Guidance Molecules
RGMA	Repulsive Guidance Molecule Co-Receptor A
BMP	Bone Morphogenetic Protein
LINC02055	Long Intergenic Non-Protein Coding RNA 2055
RPS6	Ribosomal Protein S6
TMEM132C	Transmembrane Protein 132C
GOLGA8B	Golgin A8 Family Member B
TRP	Transient Receptor Potential
TRPM	Melastatin Transient Receptor Potential
ncRNA	Non-Coding RNA
BCHE	Butyrylcholinesterase
ACHE	Acetylcholinesterase
BCL6	B-Cell Lymphoma 6

## References

[1] Talbot K. (2009). Motor neuron disease: The bare essentials. Pract. Neurol..

[2] Van Es M.A., Hardiman O., Chio A., Al-Chalabi A., Pasterkamp R.J., Veldink J.H., Van den Berg L.H. (2017). Amyotrophic lateral sclerosis. Lancet.

[3] Swinnen B., Robberecht W. (2014). The phenotypic variability of amyotrophic lateral sclerosis. Nat. Rev. Neurol..

[4] Sabatelli M., Marangi G., Conte A., Tasca G., Zollino M., Lattante S. (2016). New ALS-related genes expand the spectrum paradigm of amyotrophic lateral sclerosis. Brain Pathol..

[5] Chia R., Chiò A., Traynor B.J. (2018). Novel genes associated with amyotrophic lateral sclerosis: Diagnostic and clinical implications. Lancet Neurol..

[6] Brooks B.R., Miller R.G., Swash M., Munsat T.L. (2000). El Escorial revisited: Revised criteria for the diagnosis of amyotrophic lateral sclerosis. Amyotroph. Lateral Scler. Other Mot. Neuron Disord..

[7] Van Steen K., Moore J. (2019). How to increase our belief in discovered statistical interactions via large-scale association studies?. Hum. Genet..

[8] Niel C., Sinoquet C., Dina C., Rocheleau G. (2015). A survey about methods dedicated to epistasis detection. Front. Genet..

[9] Efron B., Hastie T. (2016). Computer Age Statistical Inference.

[10] Park M.Y., Hastie T. (2008). Penalized logistic regression for detecting gene interactions. Biostatistics.

[11] Wang S., Nan B., Rosset S., Zhu J. (2011). Random lasso. Ann. Appl. Stat..

[12] Schymick J.C., Scholz S.W., Fung H.C., Britton A., Arepalli S., Gibbs J.R., Lombardo F., Matarin M., Kasperaviciute D., Hernandez D.G. (2007). Genome-wide genotyping in amyotrophic lateral sclerosis and neurologically normal controls: First stage analysis and public release of data. Lancet Neurol..

[13] Hastie T., Tibshirani R., Wainwright M. (2019). Statistical Learning with Sparsity: The Lasso and Generalizations.

[14] Agresti A. (2015). Foundations of Linear and Generalized Linear Models.

[15] Yang Y., Zou H. (2015). A fast unified algorithm for solving group-lasso penalize learning problems. Stat. Comput..

[16] Meier L., Van De Geer S., Bühlmann P. (2008). The group lasso for logistic regression. J. R. Stat. Soc. Ser. B Stat. Methodol..

[17] Lim M., Hastie T. (2015). Learning interactions via hierarchical group-lasso regularization. J. Comput. Graph. Stat..

[18] Park H., Imoto S., Miyano S. (2015). Recursive random lasso (RRLasso) for identifying anti-cancer drug targets. PLoS ONE.

[19] Kim Y., Hao J., Mallavarapu T., Park J., Kang M. (2019). Hi-lasso: High-dimensional lasso. IEEE Access.

[20] Hinrichs A.S., Raney B.J., Speir M.L., Rhead B., Casper J., Karolchik D., Kuhn R.M., Rosenbloom K.R., Zweig A.S., Haussler D. (2016). UCSC data integrator and variant annotation integrator. Bioinformatics.

[21] McLaren W., Gil L., Hunt S.E., Riat H.S., Ritchie G.R., Thormann A., Flicek P., Cunningham F. (2016). The ensembl variant effect predictor. Genome Biol..

[22] Ward L.D., Kellis M. (2012). HaploReg: A resource for exploring chromatin states, conservation, and regulatory motif alterations within sets of genetically linked variants. Nucleic Acids Res..

[23] Boyle A.P., Hong E.L., Hariharan M., Cheng Y., Schaub M.A., Kasowski M., Karczewski K.J., Park J., Hitz B.C., Weng S. (2012). Annotation of functional variation in personal genomes using RegulomeDB. Genome Res..

[24] Machiela M.J., Chanock S.J. (2015). LDlink: A web-based application for exploring population-specific haplotype structure and linking correlated alleles of possible functional variants. Bioinformatics.

[25] Sherry S.T., Ward M., Sirotkin K. (1999). dbSNP—Database for single nucleotide polymorphisms and other classes of minor genetic variation. Genome Res..

[26] Carithers L.J., Ardlie K., Barcus M., Branton P.A., Britton A., Buia S.A., Compton C.C., DeLuca D.S., Peter-Demchok J., Gelfand E.T. (2015). A novel approach to high-quality postmortem tissue procurement: The GTEx project. Biopreserv. Biobank..

[27] Uhlén M., Fagerberg L., Hallström B.M., Lindskog C., Oksvold P., Mardinoglu A., Sivertsson Å., Kampf C., Sjöstedt E., Asplund A. (2015). Tissue-based map of the human proteome. Science.

[28] Meinshausen N., Meier L., Bühlmann P. (2009). *p*-values for high-dimensional regression. J. Am. Stat. Assoc..

[29] Laird N.M., Lang C. (2011). The Fundamentals of Modern Statistical Genetics.

[30] Sha Q., Zhang Z., Schymick J.C., Traynor B.J., Zhang S. (2009). Genome-wide association reveals three SNPs associated with sporadic amyotrophic lateral sclerosis through a two-locus analysis. BMC Med. Genet..

[31] Pan W. (2010). Statistical tests of genetic association in the presence of gene-gene and gene-environment interactions. Hum. Hered..

[32] Macintyre G., Bailey J., Haviv I., Kowalczyk A. (2010). is-rSNP: A novel technique for in silico regulatory SNP detection. Bioinformatics.

[33] Han F., Pan W. (2012). A composite likelihood approach to latent multivariate gaussian modeling of snp data with application to genetic association testing. Biometrics.

[34] Han F., Pan W. (2010). Powerful multi-marker association tests: Unifying genomic distance-based regression and logistic regression. Genet. Epidemiol..

[35] Audo I., Kohl S., Leroy B.P., Munier F.L., Guillonneau X., Mohand-Saïd S., Bujakowska K., Nandrot E.F., Lorenz B., Preising M. (2009). TRPM1 is mutated in patients with autosomal-recessive complete congenital stationary night blindness. Am. J. Hum. Genet..

[36] Li Z., Sergouniotis P.I., Michaelides M., Mackay D.S., Wright G.A., Devery S., Moore A.T., Holder G.E., Robson A.G., Webster A.R. (2009). Recessive mutations of the gene TRPM1 abrogate ON bipolar cell function and cause complete congenital stationary night blindness in humans. Am. J. Hum. Genet..

[37] Thameem F., Puppala S., Arar N.H., Blangero J., Duggirala R., Abboud H.E. (2011). Genetic variants in Transient Receptor Potential cation channel, subfamily M 1 (TRPM1) and their risk of albuminuria-related traits in Mexican Americans. Clin. Chim. Acta.

[38] Okumus S., Demiryürek S., Gürler B., Coskun E., Bozgeyik İ., Oztuzcu S., Kaydu E., Celik O., Erbagcı İ., Demiryürek A.T. (2013). Association transient receptor potential melastatin channel gene polymorphism with primary open angle glaucoma. Mol. Vis..

[39] Yamada Y., Yasukochi Y., Kato K., Oguri M., Horibe H., Fujimaki T., Takeuchi I., Sakuma J. (2018). Identification of 26 novel loci that confer susceptibility to early-onset coronary artery disease in a Japanese population. Biomed. Rep..

[40] Lv H., Zhang M., Shang Z., Li J., Zhang S., Lian D., Zhang R. (2017). Genome-wide haplotype association study identify the FGFR2 gene as a risk gene for acute myeloid leukemia. Oncotarget.

[41] Van Ness B., Ramos C., Haznadar M., Hoering A., Haessler J., Crowley J., Jacobus S., Oken M., Rajkumar V., Greipp P. (2008). Genomic variation in myeloma: Design, content, and initial application of the Bank on a Cure SNP Panel to detect associations with progression-free survival. BMC Med..

[42] Morton L.M., Purdue M.P., Zheng T., Wang S.S., Armstrong B., Zhang Y., Menashe I., Chatterjee N., Davis S., Lan Q. (2009). Risk of Non–Hodgkin Lymphoma Associated with Germline Variation in Genes that Regulate the Cell Cycle, Apoptosis, and Lymphocyte Development. Cancer Epidemiol. Biomark. Prev..

[43] Le Gall L., Anakor E., Connolly O., Vijayakumar U.G., Duddy W.J., Duguez S. (2020). Molecular and cellular mechanisms affected in ALS. J. Pers. Med..

[44] Stocker M., Kerschensteiner D. (1998). Cloning and tissue distribution of two new potassium channel *α*-subunits from rat brain. Biochem. Biophys. Res. Commun..

[45] Shepard A.R., Rae J.L. (1999). Electrically silent potassium channel subunits from human lens epithelium. Am. J. Physiol.-Cell Physiol..

[46] Jimenez I., Prado Y., Marchant F., Otero C., Eltit F., Cabello-Verrugio C., Cerda O., Simon F. (2020). TRPM channels in human diseases. Cells.

[47] Leader B., Leder P. (2000). Formin-2, a novel formin homology protein of the cappuccino subfamily, is highly expressed in the developing and adult central nervous system. Mech. Dev..

[48] Law R., Dixon-Salazar T., Jerber J., Cai N., Abbasi A.A., Zaki M.S., Mittal K., Gabriel S.B., Rafiq M.A., Khan V. (2014). Biallelic truncating mutations in FMN2, encoding the actin-regulatory protein Formin 2, cause nonsyndromic autosomal-recessive intellectual disability. Am. J. Hum. Genet..

[49] Mutalik S.P. (2018). Role of the Cytoskeleton in Regulating Axonal Tension and Growth Cone Traction Dynamics. Ph.D. Thesis.

[50] Sanchez-Pulido L., Ponting C.P. (2018). TMEM132: An ancient architecture of cohesin and immunoglobulin domains define a new family of neural adhesion molecules. Bioinformatics.

[51] Severyn C.J., Shinde U., Rotwein P. (2009). Molecular biology, genetics and biochemistry of the repulsive guidance molecule family. Biochem. J..

[52] Tang J., Zeng X., Li H., Ju L., Feng J., Yang J. (2021). Repulsive guidance molecule-a and central nervous system diseases. BioMed Res. Int..

[53] Munro S. (2011). The golgin coiled-coil proteins of the Golgi apparatus. Cold Spring Harb. Perspect. Biol..

[54] Sundaramoorthy V., Walker A.K., Yerbury J., Soo K.Y., Farg M.A., Hoang V., Zeineddine R., Spencer D., Atkin J.D. (2013). Extracellular wildtype and mutant SOD1 induces ER–Golgi pathology characteristic of amyotrophic lateral sclerosis in neuronal cells. Cell. Mol. Life Sci..

[55] Gonatas N., Stieber A., Mourelatos Z., Chen Y., Gonatas J., Appel S.H., Hays A., Hickey W., Hauw J. (1992). Fragmentation of the Golgi apparatus of motor neurons in amyotrophic lateral sclerosis. Am. J. Pathol..

[56] Boucher B., Jenna S. (2013). Genetic interaction networks: Better understand to better predict. Front. Genet..

[57] Szklo M., Nieto J. (2014). Epidemiology: Beyond the Basics.

[58] Johnson G., Moore S.W. (2012). Why has butyrylcholinesterase been retained? Structural and functional diversification in a duplicated gene. Neurochem. Int..

[59] Kozhemyakin M., Rajasekaran K., Kapur J. (2010). Central cholinesterase inhibition enhances glutamatergic synaptic transmission. J. Neurophysiol..

[60] Haidet-Phillips A.M., Hester M.E., Miranda C.J., Meyer K., Braun L., Frakes A., Song S., Likhite S., Murtha M.J., Foust K.D. (2011). Astrocytes from familial and sporadic ALS patients are toxic to motor neurons. Nat. Biotechnol..

[61] Barbour B., Brew H., Attwell D. (1988). Electrogenic glutamate uptake in glial cells is activated by intracellular potassium. Nature.

[62] Allman D., Jain A., Dent A., Maile R.R., Selvaggi T., Kehry M.R., Staudt L.M. (1996). BCL-6 expression during B-cell activation. Blood.

[63] Arlotta P., Molyneaux B.J., Chen J., Inoue J., Kominami R., Macklis J.D. (2005). Neuronal subtype-specific genes that control corticospinal motor neuron development in vivo. Neuron.

[64] Wang X.S., Simmons Z., Liu W., Boyer P.J., Connor J.R. (2006). Differential expression of genes in amyotrophic lateral sclerosis revealed by profiling the post mortem cortex. Amyotroph. Lateral Scler..

[65] Zhou L., Zhang C.L., Messing A., Chiu S.Y. (1998). Temperature-sensitive neuromuscular transmission in Kv1. 1 null mice: Role of potassium channels under the myelin sheath in young nerves. J. Neurosci..

[66] Wu T.T., Chen Y.F., Hastie T., Sobel E., Lange K. (2009). Genome-wide association analysis by lasso penalized logistic regression. Bioinformatics.

[67] Yang C., Wan X., Yang Q., Xue H., Yu W. (2010). Identifying main effects and epistatic interactions from large-scale SNP data via adaptive group Lasso. BMC Bioinform..

[68] Shi W., Lee K.E., Wahba G. (2007). Detecting disease-causing genes by LASSO-Patternsearch algorithm. BMC Proc..

[69] Wu C.H., Fallini C., Ticozzi N., Keagle P.J., Sapp P.C., Piotrowska K., Lowe P., Koppers M., McKenna-Yasek D., Baron D.M. (2012). Mutations in the profilin 1 gene cause familial amyotrophic lateral sclerosis. Nature.

[70] Hop P.J., Zwamborn R.A., Hannon E., Shireby G.L., Nabais M.F., Walker E.M., van Rheenen W., van Vugt J.J., Dekker A.M., Westeneng H.J. (2022). Genome-wide study of DNA methylation shows alterations in metabolic, inflammatory, and cholesterol pathways in ALS. Sci. Transl. Med..

[71] Gu D., Ou S., Tang M., Yin Z., Wang Z., Liu G. (2021). Trauma and amyotrophic lateral sclerosis: A systematic review and meta-analysis. Amyotroph. Lateral Scler. Front. Degener..

