# Supplementary figures and images for: Selecting Genetic Variants and Interactions Associated with Amyotrophic Lateral Sclerosis: A Group LASSO Approach
Source: J Pers Med. 2022 Aug 19;12(8):1330. doi: 10.3390/jpm12081330 (PMC9410070; doi:10.3390/jpm12081330)

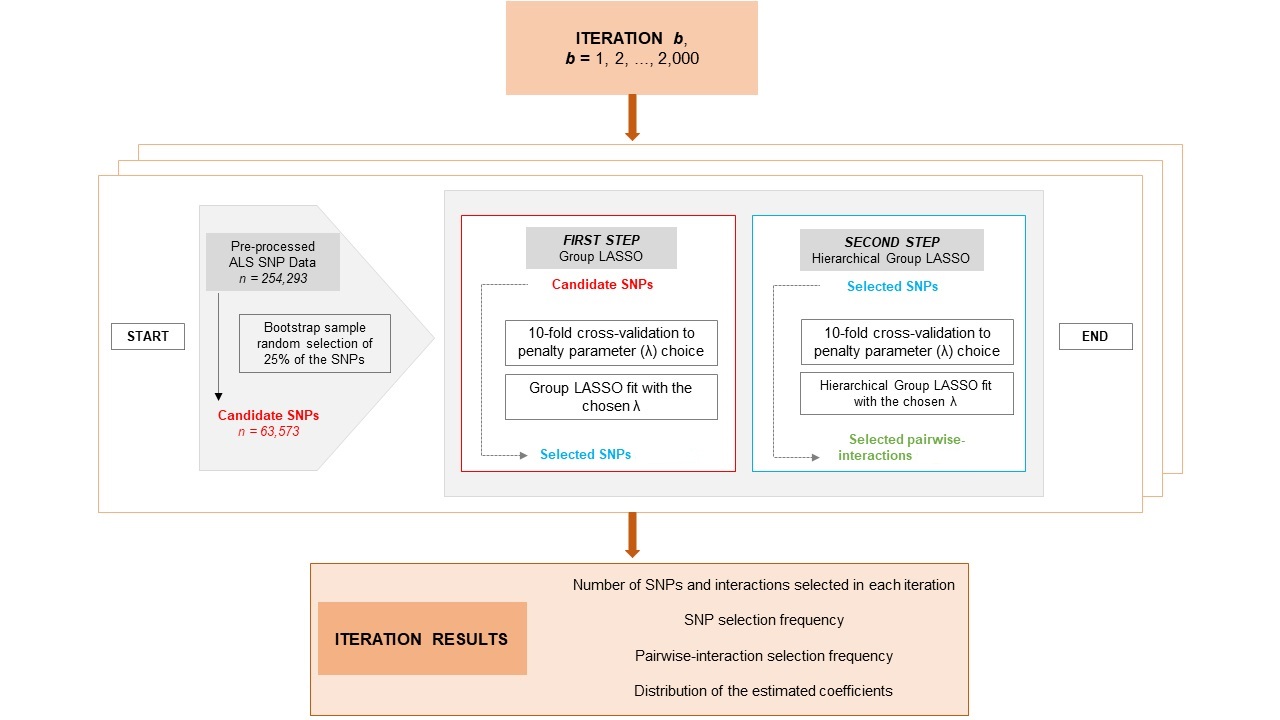

Supplement: Supplementary file 1 [file jpm-12-01330-s001.zip › supp_fig1.jpg]

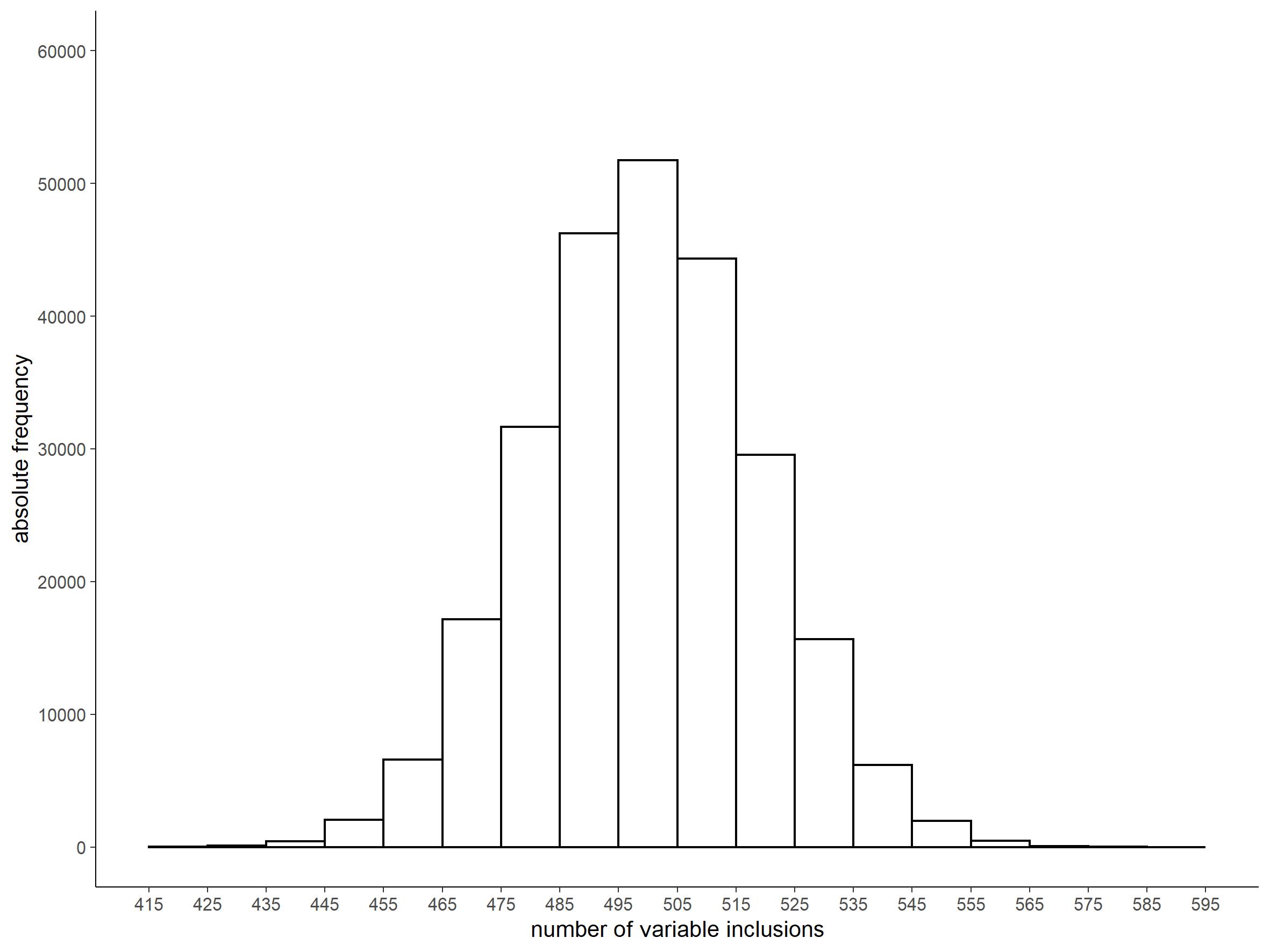

Supplement: Supplementary file 1 [file jpm-12-01330-s001.zip › supp_fig2.jpeg]

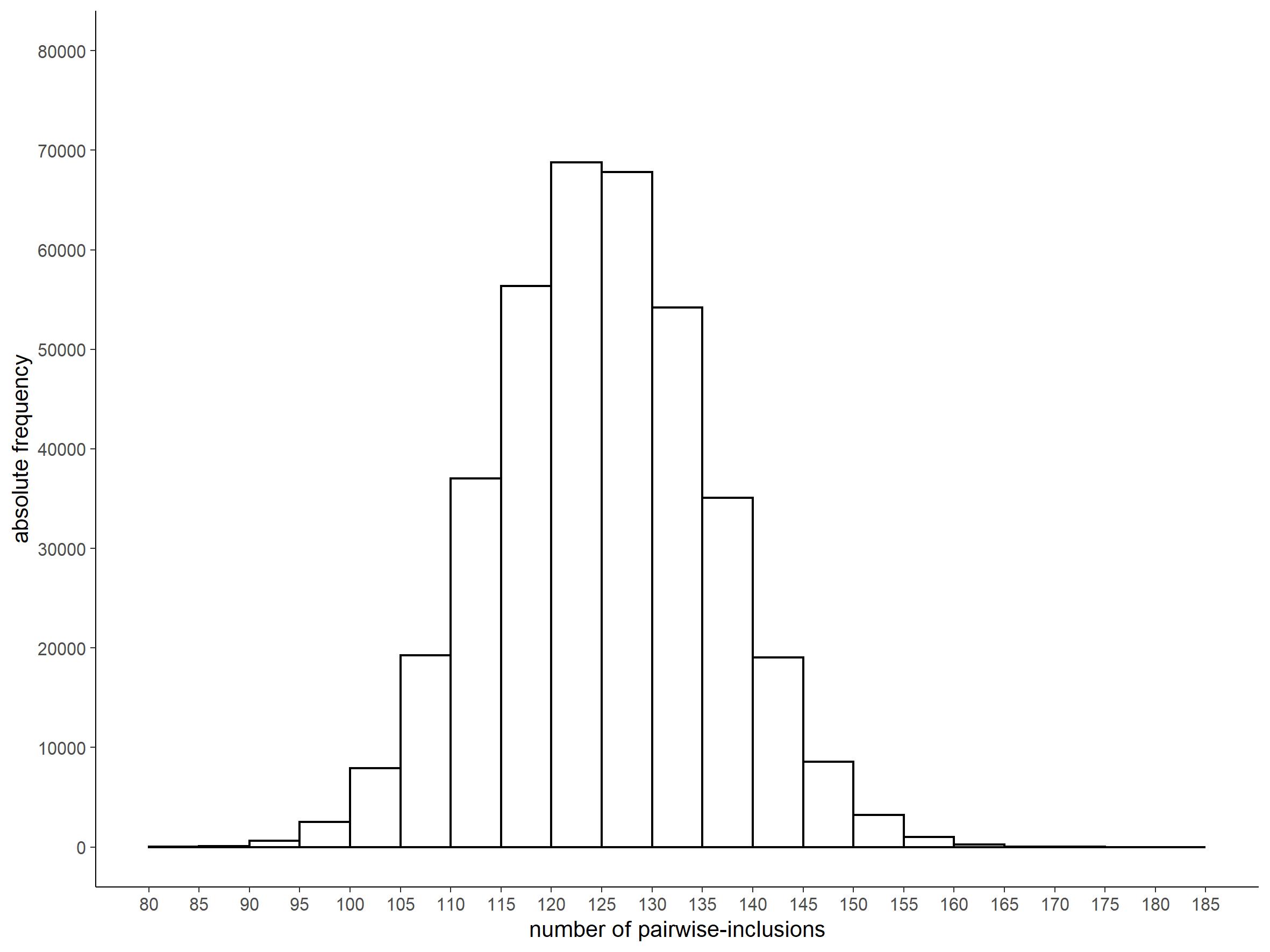

Supplement: Supplementary file 1 [file jpm-12-01330-s001.zip › supp_fig3.jpeg]

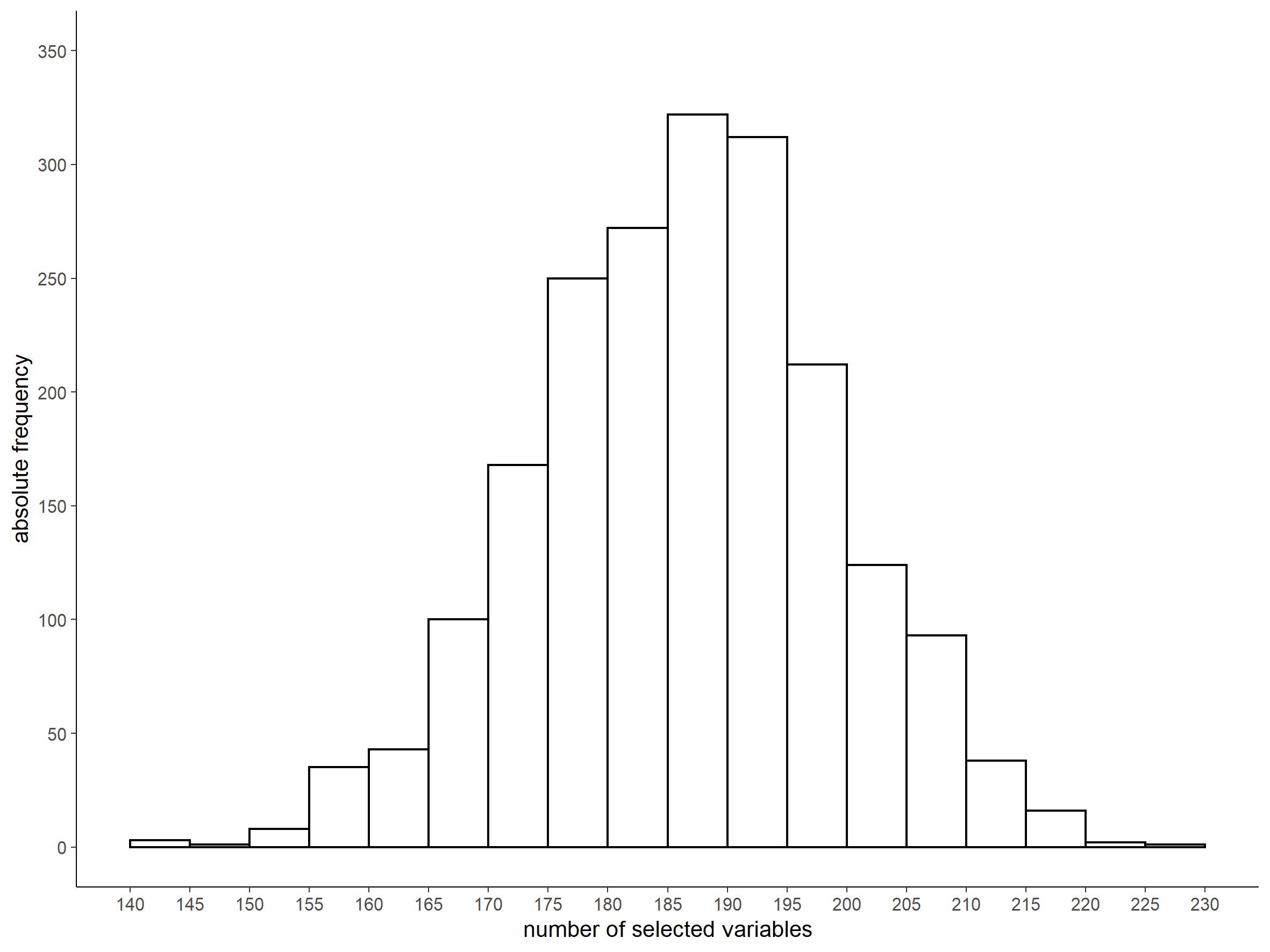

Supplement: Supplementary file 1 [file jpm-12-01330-s001.zip › supp_fig4.jpeg]

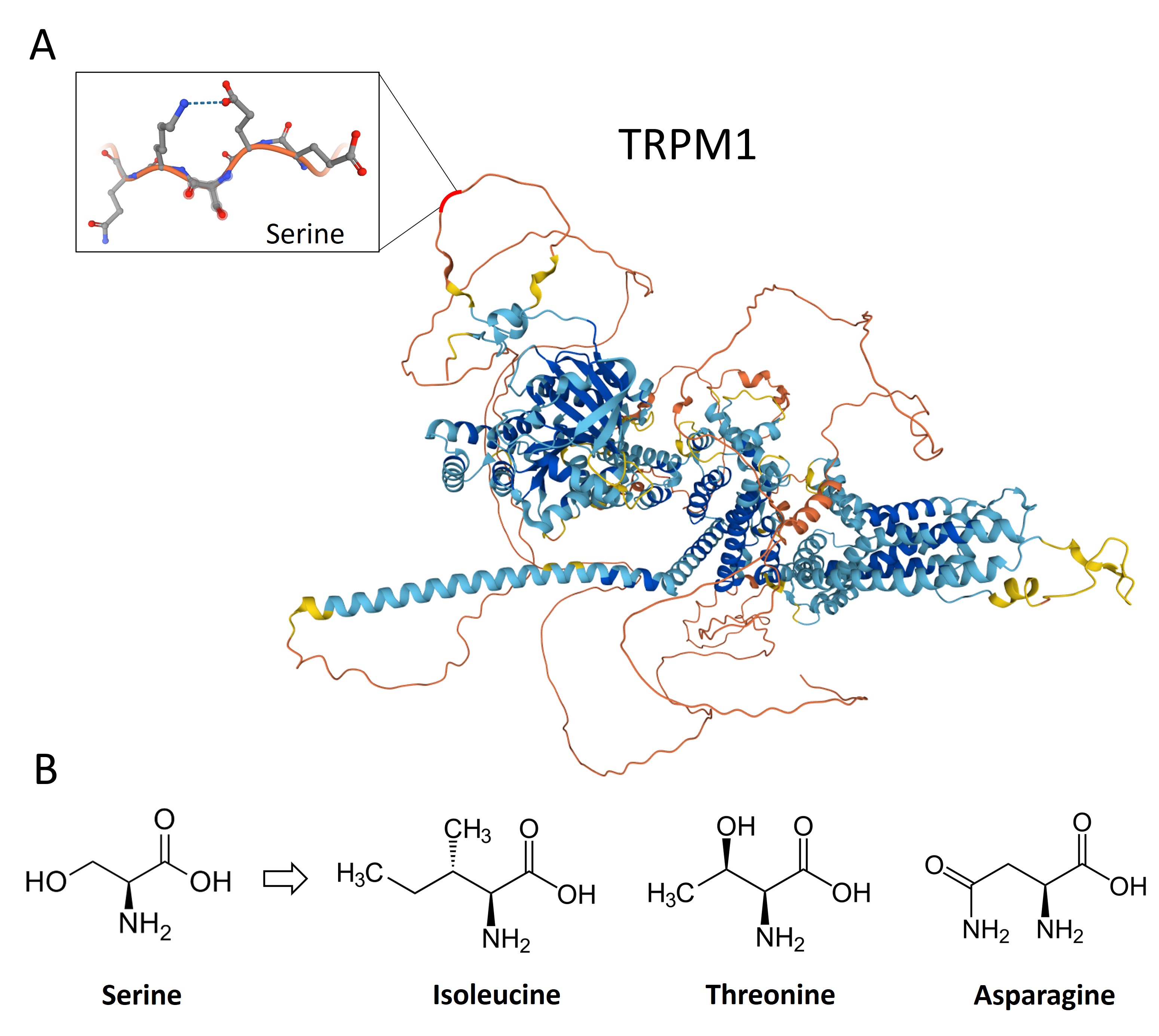

Supplement: Supplementary file 1 [file jpm-12-01330-s001.zip › supp_fig5.jpg]

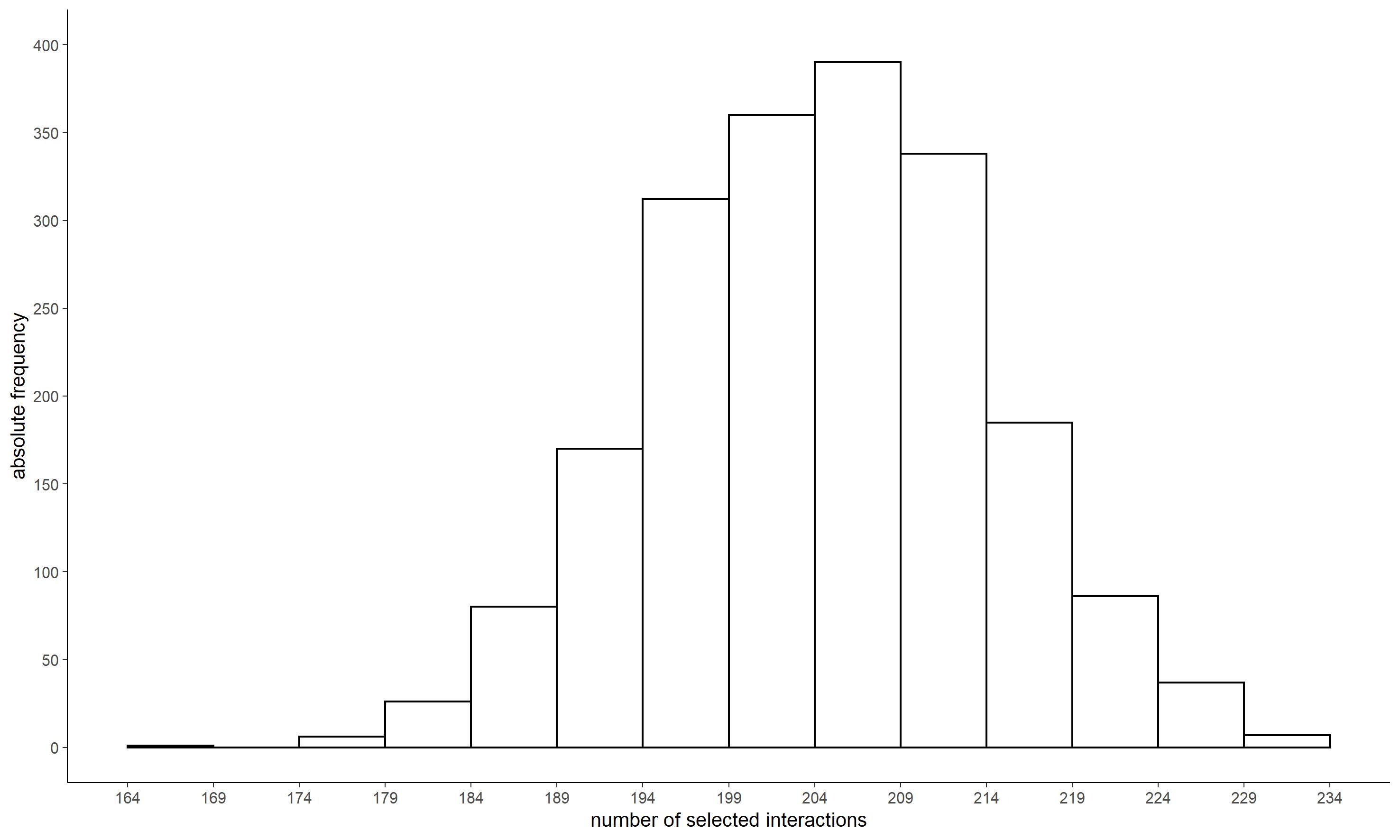

Supplement: Supplementary file 1 [file jpm-12-01330-s001.zip › supp_fig6.jpeg]
